# Obituary

**Published:** 1883-05

**Authors:** 


### OBITUARY.

---

### DEATH OF DR. WM. H. VAN BUREN.

Dr. Frank P. Foster, editor of the *New York Medical Journal*, thus announces the death of this distinguished gentleman: "William Holmes Van Buren, M.D., LL.D., was born in this city April 5th, 1819. As a surgeon, Dr. Van Buren was cool, methodical, and equal to the most trying emergencies, original and suggestive and thoroughly to be relied on in consultation—a relation in which our best men always esteemed it a pleasure to meet him. As a teacher, he was earnest, dignified, forcible, and graceful. As an author, his charm of style, his just, nay, generous, appreciation of his contemporaries, the pertinence of statements, and the aptness of his illustrations made it easy to follow him, and gave the reader the conviction from the outset that his were no idle words. As a family physician, he won not only the admiration but the warm esteem of his patients. As a man, he was honored and loved; no one could know him without these feelings, and even those who met him only casually, imbibed from him a more favorable estimate of the medical profession than they had before held. Such men the profession can ill afford to spare."

DEATH OF DR. DAVID O. FARRAND, OF DETROIT, MICH.

Probably the death of no other man could carry grief to so many as did the death of Dr. Farrand. The rich and the poor sought to do honor to the dead. A great and good physician, skillful of hand and kind of heart, has given his life to suffering humanity.

DEATH OF GEORGE MILLER BEARD, A.M., M.D.

At a meeting of the Medical Society of the County of New York, March 26th, Dr. A. D. Rockwell, his friend, presented the following resolution: That in the death of Dr. George M. Beard this Society and the profession at large have lost one of their most brilliant, earnest and active members. As an investigator, he was original and conscientious. As a friend, he was generous and steadfast. Exposed by his restless activity to many and peculiar attacks, he ever manifested the utmost charity and good humor. Of his worst enemies he seldom spoke a harsh and never a vindictive word.

